# Management of ERCP-Related Perforations: A Single-Center Experience

**DOI:** 10.3390/jcm14010001

**Published:** 2024-12-24

**Authors:** Nemanja Plecic, Ana Malenkovic, Aleksa Begovic, Aleksandra Pavlovic, Milutin Bulajic, Mirko Bulajic, Vladimir Đukic, Miljan Milanovic, Predrag Savic, Nikola Panic

**Affiliations:** 1 Digestive Endoscopy Department, University Clinic “Dr Dragisa Misovic-Dedinje”, 11000 Belgrade, Serbia; plecicnemanja94@gmail.com (N.P.); anamalenkovic@gmail.com (A.M.); aleksa.begovic@vinculabiotech.vom (A.B.); aleksandra.pavlovic1964@gmail.com (A.P.); milutin.bulajic@fbf-isola.it (M.B.); bulajic.mirko@gmail.com (M.B.); office@dragisamisovic.bg.ac.rs (V.Đ.); milanovic.miljan@gmail.com (M.M.); savic.predrag@gmail.com (P.S.); 2 Department of Digestive Endoscopy, Ospedale Isola Tiberina—Gemelli Isola, 00186 Rome, Italy; 3 Faculty of Medicine, University of Belgrade, 11000 Belgrade, Serbia

**Keywords:** ERPC, ERCP perforations, management, perforation treatment

## Abstract

**Background/Objectives:** Perforations represent rare but serious complications in ERCP. Although several therapeutic algorithms have been proposed to properly address these potentially life-threatening events, there is still no clear consensus on their management. We conducted a single-center retrospective study in order to assess the incidence of ERCP-related perforations and their management, as well as clinical outcomes. **Methods:** The hospital’s electronic database was searched in order to identify all the patients who developed ERCP-related perforations in the period 1 October 2018–30 June 2023. Perforations were classified according to the Stapfer classification. Conservative management included frequent abdominal examinations, the monitoring of vital signs, white blood cell count, complete bowel rest, nasogastric tube placement, and the administration of intravenous fluids and antibiotics. Endoscopic management included biliary stent placement and/or closing observed defects with clips. **Results:** We recorded eight (1.29%) cases of ERCP-related perforations out of the 619 procedures conducted. We observed six (75%) Stapfer type II and two (25%) type IV perforations. In all but one patient (87.5%), the indication for ERCP was bile duct stones. Seven patients (87.5%) were subjected to sphincterotomy (87.5%) and three (37.5%) to “pre-cuts”. All but one patient was treated conservatively (87.5%), with two of them—in which type II perforations were recognized intraprocedurally—also receiving endoscopic treatment with stent placement. On the day of ERCP, one patient with a type II perforation was operated on; suturing of the duodenum followed by duodenal exclusion was applied. Management was successful in all the patients, with a mean hospitalization time of 16.6 ± 4.78 days. **Conclusions:** Conservative and endoscopic management appear to be associated with good outcomes in Stapfer type II perforations. Nevertheless, an individual multidisciplinary approach involving endoscopists and a hepatobiliary surgeon is essential in order to properly guide the treatment.

## 1. Introduction

Endoscopic retrograde cholangio-pancreatography (ERCP) represents a combined endoscopic and fluoroscopic procedure for treating complex diseases of the biliary and pancreatic ducts [1,2]. Over the last fifty years, ERCP has developed from a diagnostic method conducted in a small number of dedicated centers to a widely spread and accepted strictly therapeutic procedure conducted worldwide. Although therapeutic ERCP is well established and proven to be safe and efficient, it is also associated with some serious complications, such as bleeding, post-ERCP pancreatitis, and perforations [1,2].

Iatrogenic perforations related to GI endoscopy were previously defined as the recognition of gas or fluids outside the GI tract or any endoscopically identified definite visible sign of perforation during or in the time related to endoscopy [3,4,5]. Perforations related to ERCP are rare but feared adverse events. The incidence of ERCP-related duodenal perforations is reported to range from 0.08% to 1.6% [3,4,5] and is associated with several risk factors, such as sphincterotomy, the long duration of the procedure, high age, sphincter of Oddi dysfunction, and pre-cuts [5]. ERCP-related perforations are reported to be associated with high morbidity and mortality rates, varying from 8% to even 23% [5]. Several mechanisms could be responsible for perforations during ERCP: (1) luminal perforation by the endoscope, which usually results in intraperitoneal perforation; (2) the extension of a sphincterotomy beyond the intramural portion of the bile or pancreatic duct with retroperitoneal leakage; (3) the extramural passage of guidewires or the migration of stents (6). Having this in mind, it is clear that not all ERCP-related perforations have the same clinical course and that the mechanism of iatrogenic injury has a great influence on the patient’s prognosis. Furthermore, mortality also appears to be related to late diagnosis and treatment [6].

Although several therapeutic algorithms (surgical, endoscopic, and medical management strategies) have been proposed to properly address this potentially life-threatening event, there are still no evidence-based strategies to guide clinicians. Apart from the relatively low incidence of this type of complication, the potential explanation for a lack of clear treatment strategy could be found in the heterogeneity of patients affected and the type and extension of injuries incurred [7,8]. Additionally, the outcome of the treatment is determined not only by the therapeutic strategy applied but also by several other factors, including comorbidities, the clinical status of the patient, the size and location of the perforation, radiographic imaging findings, and the interval between the perforation and the initiation of the therapy.

We have conducted a study to assess the incidence of ERCP-related perforations in our endoscopy unit, as well as to evaluate the effectiveness of several treatment approaches applied.

## 2. Materials and Methods

A single-center retrospective study was conducted at University Clinic “Dr Dragisa Misovic-Dedinje”, Belgrade, Serbia. The hospital’s electronic database was searched in order to identify all the patients who developed ERCP-related perforations in the period from 1 October 2018 to 30 June 2023. All the procedures were conducted by one experienced endoscopist. However, the number of ERCPs conducted yearly during the period the study refers to significantly varied due to the COVID-19 pandemic. Perforations were defined as previously described [9,10,11,12,13,14]. They were detected both during the procedure and post-procedurally. Intraprocedural perforations were diagnosed based on the direct visualization of a luminal defect or fluoroscopic findings demonstrating the extravasation of contrast media, the extraluminal passage of a guidewire, and/or intra- or retroperitoneal gas. Post-procedural perforations were diagnosed according to abdominal CT or intraoperative findings.

The demographic, procedural, laboratory, radiologic, and clinical data were collected from the hospital’s electronic database and subsequently analyzed.

The operator who performed the ERCP determined the perforation type based on procedural details and findings on imaging. Perforations were classified according to the classification system proposed by Stapfer et al., which incorporates the mechanism, location, and severity of perforations as predictors of surgical intervention (Table 1) [9]. Stapfer I perforations refer to endoscopy-related lateral or medial duodenal wall perforations. Stapfer II perforations represent periampullary perforations related to sphincterotomy. Stapfer III perforations encompass ductal or duodenal perforations caused by endoscopic instruments, while Stapfer IV perforations are guidewire-related perforations with the presence of retroperitoneal gas on imaging.

When a perforation was recognized and suspected, especially in patients with post-ERCP abdominal pain unresponsive to analgesics, an oral contrast abdominal CT scan was performed in all the patients. Subsequently, the decision on the management strategy was made by a multidisciplinary team including an endoscopist, a radiologist, and a hepato-pancreato-biliary (HPB) surgeon. Conservative management included frequent abdominal examination, the monitoring of vital signs and white blood cell count, complete bowel rest, and nasogastric tube placement, as well as the administration of intravenous fluids and antibiotics. If surgery was indicated, the decision on the surgical approach was made by the HPB surgeon with the consent of other members of the multidisciplinary team.

Endoscopic management included biliary stent placement and/or closing observed defects with clips and was decided on by the endoscopist during the procedure.

Collected data were analyzed using descriptive statistics.

## 3. Results

We recorded eight (1.29%) cases of ERCP-related perforations out of 619 procedures conducted over a 5-year period. Patient demographics, procedure characteristics, and types of perforations are reported in Table 2. We observed six (75%) Stapfer type II and two (25%) type IV perforations. No Stapfer type I or Stapfer III perforations were observed. In all but one patient (87.5%), the indication for ERCP was bile duct stones. In one patient indication for ERCP was plastic stent placement and biliary drainage as a part of palliative treatment for pancreatic cancer. Seven patients (87.5%) were subjected to sphincterotomy and three (37.5%) to “pre-cuts”. In five patients (62.5%), perforations were detected intraprocedurally or within 24 h.

**Figure 1:**
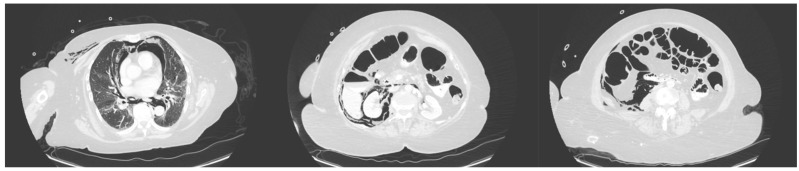
The presence of pneumoretroperitoneum, pneumoperitoneum and pneumomediastinum on CT imaging in a patient with an ERCP-associated Stapfer II perforation.

Table 3 reports the clinical, laboratory, and imaging characteristics of the cases included. All the patients presented with abdominal pain. The mean CRP value within the first 24 h upon perforation was 137.2 mg/L, and the mean WBC count was 10.8 × 10^9^/L. Retroperitoneal air on abdominal CT was observed in all the patients. Pneumoperitoneum was observed in five (62.5%) of the patients, while retroperitoneal extravasations of contrast media were detected in three (37.5%). Retroperitoneal liquid was detected in five (62.5%) patients. Figure 1 depicts the presence of pneumoretroperitoneum, pneumoperitoneum and pneumomediastinum on CT imaging in a patient with an ERCP-associated Stapfer II perforation on CT imaging in a patient with an ERCP-associated Stapfer II perforation.

**Figure 2:**
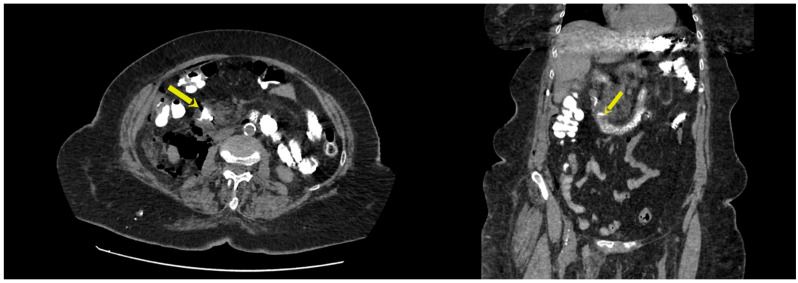
The results of a CT scan with oral contrast in a patient with a Stapfer II perforation treated intraprocedurally with plastic stent placement and endoclips. Yellow arrows point to the papillary region depicting lack of contrast extravasation.

Table 4 reports patient management and associated outcomes. All but one patient was treated conservatively (87.5%), with two of them—in which type II perforations were recognized during the procedure—also undergoing endoscopic treatment with stent placement. On the day of ERCP, one patient with a type II perforation was operated on. Suturing of the duodenum, followed by the closure of the pylorus through gastrotomy and gastrojejunostomy at the site of the gastrotomy, was applied. Management was successful in all the patients, with a mean hospitalization time of 16.6 ± 4.78 days. Figure 2 depicts the results of a CT scan with oral contrast in a patient with a Stapfer II perforation treated intraprocedurally with plastic stent placement and endoclips.

## 4. Discussion

We report the conservative and endoscopic treatments to be efficient in at least some types of ERCP-related perforations, namely, Stapfer type II.

ERCP-related perforations are one of the most feared complications of ERCP. The incidence has initially been reported to range from 0.08% to 1.6% [3,4,5]. Vezakis et al. [15] conducted a systematic review including 18 studies, reporting a pooled incidence of ERCP-related perforations of 0.39% (95% CI: 0.34–0.69) [15]. Another systematic review of 10 studies conducted by Cirrochi et al. [16] reported the pooled ERCP-related perforation rate to be 0.6%, ranging from 0.08% to 2.28%. Taking into consideration both of these literature reviews, it is noticeable that higher perforation rates are reported in individual studies in which a lower number of ERCPs was carried out. This potentially points out the experience of an endoscopic team as a pivotal factor in avoiding this type of complication. Our series on 619 consecutive ERCPs resulted in a perforation rate of 1.29%, which is comparable with a similarly sized series available in the literature. We also report 0% mortality. Conversely, pooled mortality in available systematic reviews is reported to be up to 8% [15]. However, all the perforations included in our series were Stapfer II and Stapfer IV. Although Stapfer II perforations were also observed most frequently among the studies included in the named systematic reviews, resulting in pooled incidences of 46% [15] and 68% [16], they also observed a significant number of Stapfer type I perforations, accounting for 25% [15] and 17.7% [16]. This explains the observed discrepancy in mortality, as Stapfer I perforations are associated with the highest mortality rates [17]. An important factor influencing the outcome of the treatment is the timely recognition of the perforation. Vezakis et al. [15] reported the pooled proportion of perforations recognized during ERCP to be 73%. However, in a systematic review by Cirrochi et al. [16], the diagnosis of perforation was made during the ERCP in only 36.4% of patients. We recognized ERCP-related perforations during the procedure in two out of eight patients (25%). Nevertheless, this did not significantly influence the mortality rate in our series, as treatment was successful in all of the patients affected by this adverse event. Still, 75% of perforations in our study were recognized during the first 24 h, allowing the treatment to be initiated during that timeframe. Borazan et al. [17] conducted a study comparing the outcomes in patients associated with ERCP-related perforations in relation to the time of diagnosis, reporting significant differences in mortality between the patients diagnosed during the first 24 h and beyond. Thus, it can be concluded that watchful follow-up, especially during the first 24 h after the procedure, is essential in initiating timely and efficient treatment.

Initial publications identified “pre-cut” fistulotomy and the intramural injection of contrast medium to be procedure-related risk factors for perforations during ERCP [15]. Furthermore, patients with sphincter of Oddi dysfunction, dilated common bile duct, and Billroth II gastrectomy have also been identified to be more prone to developing perforations [18]. Nevertheless, in a study conducted by Enns et al. [19], pre-cuts did not reach statistical significance as a risk factor. In our study, three out of eight perforations that occurred were associated with pre-cuts. Although our series is too small to significantly influence decision making, it is reasonable to believe that cutting through the duodenal wall increases the risk of perforation. Taking into consideration all available evidence, in their position statement on the diagnosis and management of iatrogenic endoscopic perforations, the ESGE suggested that ERCP in the setting of a papillary lesion, a dilated common bile duct, sphincter of Oddi dysfunction, or when ERCP involves sphincterotomy, precut sphincterotomy, or biliary stricture dilation should be considered to carry increased risk for iatrogenic perforation [20]. Having that in mind, the risk of perforation in the presence of named risk factors should be additionally emphasized in the ERCP training curriculum.

The first and most important dilemma, after ERPC-related perforation is recognized, is the method of treatment. Initial guidelines addressing this issue published several decades ago suggest that endoscopy-related perforations should be referred for immediate surgery [21]. The authors emphasized the clinical challenge of identifying patients who will not respond to standard conservative measures early, leading to the development of life-threatening conditions such as sepsis. Nevertheless, in time, evidence began to mount suggesting that conservative treatment is associated with good results in at least some of the patients with ERCP-related perforations [22]. Risk factors for the failure of conservative treatment, such as the inadequate drainage of bile into the duodenum, have been identified, leading to modifications of treatment algorithms [22]. Some endoscopists recommended placing a nasobiliary tube after a perforation in order to minimize retroperitoneal contamination. What followed was a pivotal paper published by Stapfer et al. [9], who not only confirmed that at least some ERCP-related perforations can be successfully treated conservatively but also proposed a classification based on the anatomic location, mechanism, and severity of the injury in order to predict the need for surgery [9]. However, even after introducing the Stapfer classification into clinical practice, some authors still advocated for at least duodenal diversion surgery for type II perforations [23], most reports agreed that Stapfer type II perforations can be successfully treated in a conservative manner [19]. In the years to come, several series confirmed these findings, reporting that conservative treatment is a feasible and appropriate treatment option for ERCP-related perforations, excluding Stapfer type I [24,25,26]. Furthermore, Kumbhari et al. have devised a management protocol for ERCP-related perforations [27]. The algorithm implies that Stapfer type I perforations, or endoscope-related luminal perforations, should be treated with immediate surgical management, while Stapfer type II perforations, or periampullary perforations, should be managed medically. Such an approach was officially formalized by the ESGE in its guidelines, published in 2020, suggesting nonsurgical management in the majority of ERCP-related periampullary or biliopancreatic ductal iatrogenic perforations [20]. The guideline also suggests that endoscopic closure should be considered depending on the type of iatrogenic perforation, its size, and the expertise of the endoscopist available at the center [20]. Among the tools available, through the scope (TTS) clips may be used for Stapfer types I and II perforations. However the success of this approach may be hindered by the size of the defect and its location. Furthermore, the duodenoscope elevator can affect a clip’s deployment. In order to resolve this issue, some authors have proposed the use of multiple TTS clips [28], duodenoscope-friendly clips [29], or TTS combined with endoloops [30]. In the case that Stapfer II perforations are diagnosed during the procedure, ESGE guidelines emphasize that biliary drainage is essential to preventing the leakage of bile into the perforation site [20]. For this purpose, the ESGE suggests using plastic stents, fully covered metal stents (FCSEMSs), and nasobiliary drains [20]. It has been reported that FCSEMSs perform better in comparison to nasobiliary drainage in relation to pain, length of hospital stays, and retroperitoneal abscess formation [31]. If a Stapfer II perforation is diagnosed after the procedure, it should be assessed by a CT scan with contrast administered orally to demonstrate the degree of leakage [20]. Major leaks present a clear indication for immediate surgery. Apart from major leaks, sepsis despite non-surgical treatment, the presence of peritonitis, and retroperitoneal fluid collections not amenable to percutaneous drainage are also amendable for surgical treatment [20]. In our series, our medical team did not follow any formal protocol, but the decision on the therapeutic approach was made by a multidisciplinary team based on all the available clinical data. Nevertheless, the results of our series speak in favor of the ESGE recommendations, as we found conservative and endoscopic management to be associated with good outcomes in Stapfer type II perforations. However, in addition to the authors previously addressing this important subject, we also note the indispensable role of a multidisciplinary team, including an endoscopist, surgeons, and radiologists, which should meet regularly in order to address any observed changes in patient conditions.

Our study has limitations. The results we are publishing come from a retrospective series that included a relatively small number of ERCPs, resulting in a small number of perforations available for analysis. Nevertheless, considering the limited data available on this subject, we consider our findings important, especially as they are in line with previously suggested protocols favoring a conservative therapeutic approach.

## 5. Conclusions

In conclusion, we recommend that Stapfer type II perforations should initially be managed medically. A low threshold for admission, the use of a simple algorithm that stratifies management according to the mechanism of injury, and a multidisciplinary approach to addressing patients during their admission resulted in excellent clinical outcomes with a short length of stay.

## Figures and Tables

**Table 1:** Stapfer classification of iatrogenic duodenal perforations during ERCP [9].

Type	Definition
I	Lateral or medial duodenal wall perforation, endoscope-related
II	Periampullary perforations, sphincterotomy-related
III	Ductal or duodenal perforations caused by endoscopic instruments
IV	Guidewire-related perforation with the presence of retroperitoneal gas on imaging

**Table 2:** Baseline demographic, clinical, and procedural characteristics of the study cohort.

Patient	Age	Gender	Indication for ERCP	Bile Duct Canulated	Sphincterotomy	Stone Extracted	Precut	Perforation Type
1	80	F	Bile duct stones	Yes	Yes	No	No	Stapfer 4
2	75	F	Bile duct stones	Yes	Yes	Yes	No	Stapfer 2
3	83	M	Bile duct stones	Yes	Yes	No	Yes	Stapfer 2
4	80	F	Bile duct stones	Yes	Yes	Yes	Yes	Stapfer 2
5	68	M	Bile duct stones	Yes	Yes	No	No	Stapfer 2
6	66	M	Pancreatic cancer	No	No	N/A	Yes	Stapfer 2
7	49	F	Bile duct stones	Yes	Yes	No	No	Stapfer 4
8	58	F	Bile duct stones	Yes	Yes	Yes	No	Stapfer 2

**Table 3:** Clinical, imaging, and radiological features of ERCP-related perforations.

			CT Features				Initial Lab. Analyses	
Patient	Perforation Type	Abdominal Pain	Retroperitoneal Air	Pneumoperitoneum	Contrast Leakage	Intraabdominal Liquid	CRP^®^	WBC Count^3^
1	Stapfer 4	Yes	Yes			Yes	27.5	3.8
2	Stapfer 2	Yes	Yes		Yes	Yes	5.1	4.9
3	Stapfer 2	Yes	Yes	Yes		Yes		
4	Stapfer 2	Yes	Yes	Yes		Yes	18.6	13.7
5	Stapfer 2	Yes	Yes	Yes	Yes	Yes	65	11.7
6	Stapfer 2	Yes	Yes	Yes	Yes			
7	Stapfer 4	Yes	Yes				367	19.3
8	Stapfer 2	Yes	Yes	Yes			340	11.4
^®^= mg/L	^3^= ×10^9^/L							

**Table 4:** Perforation management and associated outcomes.

			Treatment				
Patient	Perforation Type	Time of Diagnosis	Conservative	Endoscopic	Surgery	Length of Stay	Outcome
1	Stapfer 4	later than 24 h	Yes			27	Discharged
2	Stapfer 2	Intraprocedural	Yes	Yes		15	Discharged
3	Stapfer 2	Intraprocedural	Yes	Yes		15	Discharged
4	Stapfer 2	later than 24 h	Yes			17	Discharged
5	Stapfer 2	within 24 h			Yes	13	Discharged
6	Stapfer 2	within 24 h	Yes			/	Discharged
7	Stapfer 4	later than 24 h	Yes			18	Discharged
8	Stapfer 2	within 24 h	Yes			11	Discharged

## Data Availability

The row data supporting the conclusions of this article will be made available by the authors on the request.
